# Assessment of Xsens Motion Trackers’ Accuracy to Measure Induced Vibrations During Endurance Running

**DOI:** 10.3390/jfmk11010082

**Published:** 2026-02-18

**Authors:** Chiara Martina, Andrea Appiani, Diego Scaccabarozzi

**Affiliations:** Mechanical Engineering Department, Politecnico di Milano, 20156 Milano, Italy; chiara.martina@polimi.it (C.M.); andrea.appiani@polimi.it (A.A.)

**Keywords:** human response to vibration monitoring, long-distance running, MEMS, IMU for sport applications, wearable devices, running-related injuries (RRIs), endurance training

## Abstract

**Background:** Research on vibrations induced by running has gained significant attention due to its implications for athletes’ performance, injury prevention, and overall well-being. Distance running exposes the body to repetitive impulsive forces, causing significant vibrations to travel through physiological systems and biomechanical structures. These vibrations increase fatigue and the risk of injury. Although it has gained importance, research on induced vibration during running and wearable equipment for monitoring is scarce. This study aims to evaluate the performance of a measurement system for monitoring the acceleration levels of induced vibrations during long-distance running, exploring the capability of non-invasive wearable devices to characterise vibration transmissibility and exposure. Moreover, a preliminary quantitative assessment of induced vibration levels for an indoor testing scenario is given. **Methods:** Metrological characterisation of Xsens Motion Trackers Awinda (MTw), off-the-shelf inertial magnetic motion trackers, was performed by measuring the sensors’ frequency bandwidth in a controlled environment, providing logarithmic sweep sine excitations at different levels (2 *g*, 5 *g*, 7 *g*, where *g* is meant to be the gravitational acceleration). A testing protocol for indoor testing was derived from the literature, allowing characterisation of the sensors’ behaviour in terms of vibration transmissibility and exposure detection in the intended application. Time domain and frequency domain analyses were conducted by following the ISO 2631 standard guideline for vibration exposure assessment, and measurement uncertainty was defined, either for the dynamic correction of the sensors’ frequency behaviour or for the computed time and frequency domain metrics. In this framework, a treadmill-based test was conducted. The aim was to evaluate the Xsens sensors’ performance in measuring vibration dose exposure and transmissibility. Three MTws were placed on the subject’s right tibia, back, and forehead using elastic bands. A 25-year-old female amateur runner completed a series of tests consisting of walking for 1 min at 3.5 km/h (instrumentation setup), followed by running at two speeds (8 km/h and 11 km/h) for 2–4 min per trial, with 5 min rest periods between tests. **Conclusions:** The tested measurement system showed promising results due to its capability to assess vibration exposure during sports activities, but dynamic correction was found to be mandatory for accurate vibration level assessment. The main outcome of this study is a method for characterising the accelerometers embedded in the proposed devices, along with an analysis strategy for future testing campaigns. Thanks to the portability of IMUs (inertial measurement units), this approach enables the evaluation of induced vibrations during in-field running measurements.

## 1. Introduction

Vibrations induced by running have emerged as a significant area of research due to their potential implications for runners’ performance, injury prevention, and overall well-being. The repetitive impulsive inputs introduced during the activity generate vibrations that propagate through the body, affecting various physiological systems and biomechanical structures; indeed, long-distance running can place significant stress on the lower limbs and musculoskeletal system, leading to various running-related injuries (RRIs), such as osteoarthritis, patellofemoral pain syndrome, Achilles tendinopathy and stress fractures [1,2,3,4,5]. According to literature, higher weekly training distance in male runners and a history of previous injuries are associated with a higher risk of such injuries, even though further well-designed studies are needed to better understand the risk factors for male and female runners [6,7].

The effects of long-distance running are currently being studied extensively [8,9]; however, the literature is still scarce in terms of causes of the associated injuries, which is particularly important for designing mitigation strategies and rehabilitation protocols for long-distance runners. An interesting study investigated the running-induced vibration phenomena in soft tissues [10], which are characterised by natural frequencies between 5 and 55 Hz, corresponding to the typical frequency range of the impacts generated during running [11,12,13]. While one might expect a significant increase in vibration amplitude observed in soft tissues, studies by Nigg and Boyer et al. [14,15,16] have revealed that the human body has strategies to regulate the level of vibrations; the control and the muscle tuning allow the central nervous system to adjust muscular activity, particularly near resonance conditions, to enhance the damping effect.

Additionally, Frederick and Hagy [17] demonstrated that an increase in body mass correlates with heightened vibration amplitudes. Nevertheless, the effects of prolonged exposure to phenomena typical of long-distance running have not been evaluated yet.

Many factors can impact soft-tissue vibration levels, both intrinsic, such as biomechanical and muscle patterns, and external, such as fatigue [18,19,20], footwear [21,22,23] and running speed [14,24]. Indeed, each combination of footwear and speed provides a specific impulse impact signal to the locomotor system. In this framework, it has been shown that changing the property of the shoe insole does not modify the magnitude of the impacts [25], but it modifies its dynamic behaviour [26]. Indeed, Boyer highlighted that it has a strong influence on the loading rate, which is the time derivative of the impact signal. During previous studies conducted on running speed and its consequences on the human body, it has been found that there is a relationship between speed, impact shock, and the amplitude of vibrations transmitted to soft tissues and to the head. Measurements have demonstrated that the amplitude of vibrations in soft tissues, especially in muscles, increases with increasing running speed. Regardless of the muscle analysed, the mean power increased significantly with increasing speed. This trend also applies to frequencies, which increase with running speed [24], and indicates the necessity of further investigating the prolonged exposure of the human body.

As demonstrated by the attention given to the topic, transmitted vibration on tissues and the related effects are of paramount importance in the scientific community. However, the literature about vibration dose during running is still scarce and, moreover, limited to athletes tested in a controlled environment, i.e., indoor treadmill running. A standardised testing procedure has not yet been established or published. The most commonly used techniques in the literature for measuring induced vibrations are video tracking with passive markers and tri-axial accelerometers [27,28,29].

Recent advances in wearable technology have significantly improved its quality and accessibility, allowing instrumental analysis of running to extend beyond the laboratory into clinical and outdoor real-scenario settings [30]. With their recent implementation in outdoor contexts, wearable devices show promising potential for monitoring additional parameters, such as soft-tissue vibrations during endurance running, providing insight into musculoskeletal load and injury risk. Despite widespread use of IMUs for kinematic analysis, the application of commercial wearable sensors, such as the Xsens MTw Awinda, for quantifying vibration exposure during endurance running remains largely unexplored. Most prior studies have focused on accelerometers for joint kinematics or lab-based vibration measurements, often neglecting real-world running conditions and soft-tissue dynamics. This highlights an unmet need for validating commercial IMUs in field-based vibration dose assessment, providing both practical and reproducible approaches for monitoring musculoskeletal loading in endurance athletes.

Thus, this study aims to assess the feasibility of using off-the-shelf commercial sensors for induced running vibration monitoring, to be used either in a laboratory or outdoors. Besides the portability and minimal impact on the tester, the advantage of using the selected sensor lies in its ability to collect additional information, such as the orientation angles and the gyroscope output, which can complement the acceleration level data for reconstructing the posture of the athlete during the activity. The analyses of kinematic data collected during sports activities and in running have yielded promising results, establishing a strong basis for utilising this kind of device in dynamic analyses, including outdoor environment applications [31,32]. However, the literature on the applicability of this type of sensor for vibration monitoring is scarce, and metrological characteristics related to their dynamic behaviour are not fully explored. The latter points are the major objectives of this research.

The study first provides a detailed description of the Xsens Motion Trackers Awinda (MTw) and the methodology used for data acquisition and processing (Section 2), including the metrological characterisation of the sensors, evaluation of their frequency bandwidth, and estimation of measurement uncertainty. Then, the dynamic calibration test and a preliminary indoor running monitoring testing activity are described in Section 3; these were specifically designed to assess the sensors’ capability to measure vibration transmissibility and exposure during endurance running. Time- and frequency domain analyses were performed to evaluate the vibration levels. The results of these investigations are presented and discussed in Section 4, while Section 5 outlines future research directions.

## 2. Materials and Methods

### 2.1. Xsens Motion Tracker Description

Xsens Motion Trackers Awinda (MTw), by Xsens Technologies B.V. (Enschede, The Netherlands) are miniaturised wireless inertial measurement units characterised by a rectangular case of 47 mm × 30 mm × 13 mm and a mass of 16 g, containing several micro-electromechanical systems (MEMS), such as three-axial linear accelerometers, 3D gyroscopes, 3D magnetometers, and a barometer. Table 1 shows the accelerometers’ nominal characteristics.

According to the manufacturer’s specifications, the accelerometers operate over a full-scale range of ±160 m/s^2^ with a bandwidth suitable for human motion analysis and impact-related phenomena. The devices were used within the limits specified by the manufacturer and following the recommended operating conditions [31,32].

Each MTw motion tracker is wirelessly connected to the MTw Awinda Station (Master), which uses the Awinda protocol to receive and time-synchronise data from up to 20 MTws simultaneously. Table 2 summarises the sensors’ technical specifications. Two important limitations must be underlined: the distance between the station and the trackers and the resampling of acceleration data necessary to be transmissible wirelessly.

The SDI algorithm integrated into the MTw Awinda protocol reduces the data rate; nevertheless, it can provide consistent results from previously calibrated data processing [32].

Then, as each MTw connects to the Awinda Master, the maximum update rate decreases; the maximum sampling rate available for five devices simultaneously is 120 Hz, thereby limiting the measurement frequency range to 60 Hz.

### 2.2. Transducer’s Dynamic Calibration

The inertial measurement units (IMUs) typically use a non-linear Kalman filter-based sensor fusion algorithm to estimate both orientation and gravity-compensated acceleration from raw signals acquired by tri-axial accelerometers, gyroscopes, and magnetometers. This approach provides a calibration procedure that is both necessary and sufficient for kinematic studies [33,34] but neglects the need to correct for the dynamic behaviour of the used sensors. The latter is fundamental to provide accurate, unbiased measurements of the vibration levels within the sensors’ bandwidth.

The calibration of the three-axis accelerometers of the MTw devices was carried out to verify the sensor characteristics within the range of interest before application to running; as previously stated, the bandwidth for multiple sensor acquisition is limited up to 60 Hz, in agreement with the frequency range of interest shown by the literature in previous studies [25].

**Figure 1:**
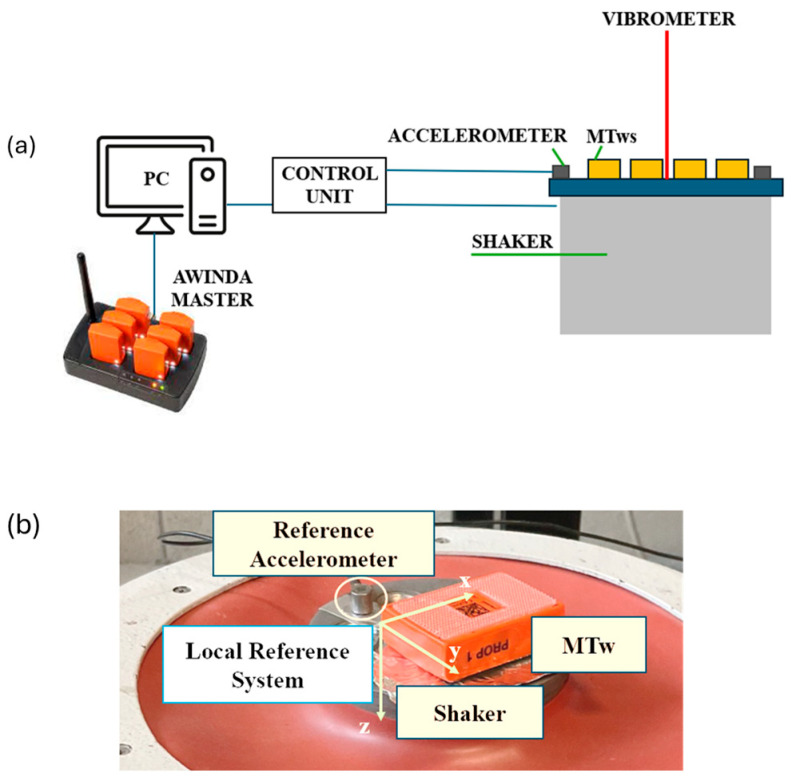
Calibration setup scheme and reference systems: (**a**) representation of the measurement chain and setup; (**b**) view of one transducer mounted on the shaker head and definition of the MTw local reference system.

The calibration setup, shown in Figure 1a, consisted of an electromagnetic shaker (TIRAvib 5010 model, by TIRA GmbH, Schalkau, Germany) as a controlled vibration source, a piezoelectric reference accelerometer (Monoaxial Endevco 27A11 manufactured by Endevco, Irvine, CA, United States of America, SN 10108, with a nominal sensitivity equal to 1.002 mV/m/s^2^) to control shaker head acceleration, and a laser vibrometer (Polytec^TM^ OFV-505 model, manufactured by Polytec GmbH, Waldbronn, Germany) to acquire the shaker head acceleration in time. The latter was used as a reference for the calibration due to its high measurement accuracy. The global reference system is also reported, as well as the local reference system of the motion trackers; both the measurement systems are shown in Figure 1b.

While the sensors were fixed during calibration, on-field use requires identifying the local reference system to evaluate accelerations in the global reference system, as suggested by ISO standards (ISO 2631-1:1997). The calibration procedure consisted of different testing activities: the shaker was set to generate a logarithmic sweep sine from 10 Hz to 55 Hz at three different acceleration levels, i.e., 2 *g*, 5 *g* and 7 *g* (*g* is meant to be the gravitational acceleration). Each acquisition direction, as defined by the reference system, was tested by aligning it with the shaker excitation direction in order to evaluate the dynamic behaviour of the sensor along the three acquisition axes. Furthermore, each acceleration level tested was repeated two times to account for measurement repeatability. The vibrometer and accelerometer signals were measured by an acquisition board (NI 9234—National Instrument) at a sampling frequency of 2048 Hz, whereas data coming from the MTws were acquired through the MT Manager software (MT Software Suite 2022.2 for Awinda), which samples the signal at 120 Hz maximum frequency. This configuration was selected because, during running tests, at least three sensors would be used to track the vibration levels.

The acquired data were analysed following the steps reported below:

1. Checking the stability of the motion trackers’ angles to guarantee the consistency of the mounting interface on the shaker;
2. Removal of the gravitational acceleration (acting along Z direction) from the measured time histories by high-pass filtering the measured acceleration (1 Hz frequency, Butterworth, 3rd order, high-pass filtering);
3. Laser vibrometer signal resampling at 120 Hz;
4. Signal synchronisation using cross-correlation between the acquired acceleration from MTws and the vibrometer.

After these post-processing steps, FFT (fast Fourier transform) was computed for both the measured velocity and acceleration, and in order to compare the measured reference and the MTws’ output, the velocity was integrated in the frequency domain (by using the *jω* method). Then, for each motion tracker, the FRF (frequency response function) was computed, considering the acceleration spectrum from the vibrometer as input (*x*(*t*)) and the acceleration measured by the motion trackers as output (*y*(*t*)). FRF was evaluated by computing the *H*_1_(*f*) estimator according to Equation (1):

(1) $$H_{1}(f)=\frac{S_{xy}(f)}{S_{xx}(f)}$$

where *S_xy_*(*f*) and *S_xx_*(*f*) are the cross-spectrum between input and output and the auto-spectrum of the input, respectively.

### 2.3. Testing Procedure and Data Analysis Techniques for Running Vibration Level Assessment

In order to assess the performance of the Xsens sensors in measuring vibration dose exposure and transmissibility, a testing procedure on a treadmill was defined. The advantage of using a treadmill (Technogym, Skillrun Live 5000 model, manufactured by Technogym S.p.A., Cesena, Italy) was that it allowed for setting the pace of the run at a constant speed. Three MTws were placed over the tested subject using elastic scratch bands; in particular, these were placed on the right tibia (right below the knee), on the back (at the level of the hips) and on the forehead. These positions were selected according to previous literature studies [27]. Two running speeds (i.e., 8 km/h and 11 km/h) and durations (i.e., 2 min or 4 min) were used. Below is a summary of the test procedure:

- Walking for 1 min at 3.5 km/h on the treadmill, to set the instrumentation—referred to as Test 0.
- Running for 4 min at 8 km/h—referred to as Test 1.
- Running for 4 min at 11 km/h—referred to as Test 2.
- Running for 2 min at 8 km/h—referred to as Test 3.
- Running for 2 min at 11 km/h—referred to as Test 4.

**Figure 2:**
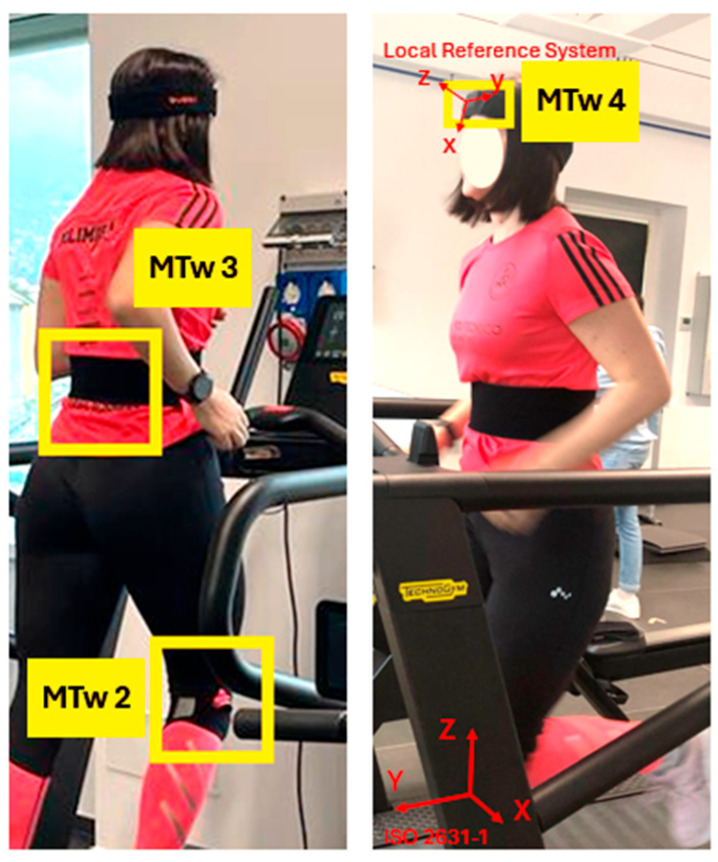
Views of the MTw mounting: the positions of MTws are highlighted in yellow boxes, whereas the ISO 2631-1 and local reference system for MTw4 are shown in red colour.

After each test, in agreement with what has been reported in [27], the subject rested for 5 min—a sufficient time for the heart rate to return to resting levels. A 25-year-old female subject (height = 1.64 m, weight = 63 kg) was selected for the previously defined testing procedure. The subject is an amateur runner (10 km distance twice a week), without any history of injuries. In Figure 2, MTw positioning on the subject and the reference systems (ISO standard and local one) are shown. Although no standardised methods for this type of testing are currently available in the literature [10], according to [27], the transmissibility of vibrations through the body can be evaluated by positioning the accelerometers on the subject, focusing on the tibia (right below her knee), on the back (at the level of the hips) and on the forehead.

**Figure 3:**
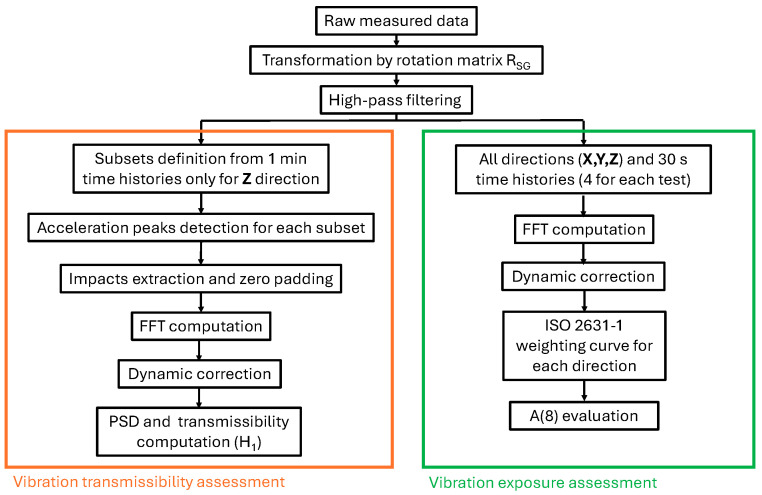
Processing flowcharts for vibration transmissibility and exposure assessment.

The acquired raw signals from the MTws followed a customised processing procedure depending on the analysis objective, as shown in Figure 3.

The acquired acceleration signals were transformed into the global reference system for the vibration exposure assessment (the Z direction is aligned with the vertical direction) by a rotation matrix $R_{GS}$, which is defined in Equation (2). For that purpose, the measured angle trends by MTws were used. In Equation (2), *ψ*, *θ*, and *φ* are the yaw, the pitch, and the roll angles, respectively.

(2) $$R_{GS}={R^{Z}}_{\psi }{R^{Y}}_{\theta }{R^{X}}_{\varphi }=\;\left[\begin{array}{ccc} cos\psi & -sin\psi & 0\\ sin\psi & cos\psi & 0\\ 0 & 0 & 1 \end{array}\right]\left[\begin{array}{ccc} cos\theta & 0 & sin\theta \\ 0 & 1 & 0\\ -sin\theta & 0 & cos\theta \end{array}\right]\left[\begin{array}{ccc} 1 & 0 & 0\\ 0 & cos\varphi & -sin\varphi \\ 0 & sin\varphi & cos\varphi \end{array}\right]$$

Moreover, high-pass filtering was applied (Butterworth, 3rd order, cutoff frequency 0.5 Hz) to remove the gravitational acceleration component, which is not of interest for the vibration transmissibility and exposure analyses.

In order to evaluate the vibration transmissibility, measured time histories were analysed by considering one-minute sub-histories for each acquisition, considering only the Z direction (from the ISO 2631-1 reference system), as the literature shows that it is the most relevant in terms of measured vibration amplitudes. The same approach can also be applied to the other axes.

Subsequently, for each selected sub-history, acceleration peaks were detected by using a numerical code developed in MATLAB 2024b. Each peak was then used to trigger a selected portion of the signal (24 samples, i.e., about 200 ms) into subsets, allowing for averaging of the analysed data. In order to improve the frequency resolution for the subsequent frequency domain analyses, zero-padding [25] was implemented (i.e., 200 points before and after the extracted ones), resulting in a frequency domain resolution of about 0.283 Hz.

Once the subsets were created, FFTs were computed to obtain the spectra: the FFTs were performed for every single subset, and dynamic correction was applied using the correction function obtained during the calibration process. PSDs (power spectral densities) and *H_1_* were then computed by averaging the spectral quantities of the subsets, i.e., computed auto-spectra and cross-spectra, to complete the transmissibility assessment analysis.

Vibration exposure assessment considers the daily vibration exposure coefficient, expressed as the equivalent continuous acceleration over an 8 h period. It quantifies daily vibration exposure in compliance with the ISO 2631-1:1997 standard [35]. This type of evaluation, commonly used to assess whole-body vibration, has previously been applied in sports contexts [36,37,38]. In this research, due to the absence of a standardised parameter for predicting running-related injuries (RRIs) in the existing literature, the daily vibration exposure analysis was used.

The data processing procedure started with the acquired raw data, considering subsets of 30 s duration for each tested condition, to assess repeatability of the measured statistics. FFT was computed for each subset, and dynamic correction and ISO 2631 weighting curves (two different curves for lateral and vertical excitations) were applied in the frequency domain for each vibration direction. From the obtained weighted spectra, RMS acceleration values were computed as

(3) $${RMS}_{i\;}=\;\sqrt{\sum_{k=1}^{N_{s}}\frac{{\left|G_{k,i}\right|}^{2}}{2}}\;$$

where “*i*” is the considered direction, “*_k_*” is the *k*-th component of the spectral amplitudes, *G_k,i_* is the *k*-th double-sided spectral component from the FFT, and *N_s_* is half of the overall spectral components. Having obtained the RMS values along the three measurement directions, the frequency-weighted RMS acceleration *a_w_* was computed as

(4) $$a_{w}=\sqrt{{{RMS}_{X}}^{2}+{{RMS}_{Y}}^{2}+{{RMS}_{Z}}^{2}}$$

Furthermore, the *A*(8) value, which reflects the intensity of vibration to which a subject is exposed, was computed. This metric scales *a_w_* by the square root of the ratio between the actual exposure duration and a reference period of 8 h, as defined by the ISO 2631-1 standard:

(5) $$A\left(8\right)=a_{w}\cdot \sqrt{\frac{T_{i}}{T_{0}}}$$

*T_i_* represents the actual exposure duration in seconds, *a_w_* is the frequency-weighted RMS acceleration, and *T*_0_ is the reference duration. It has to be noted that the standard specifies that the comfort threshold for whole-body vibration exposure is 2 m/s^2^.

The applied processing methods allow for a standardised assessment of exposure levels during running, considering both the location of the measured vibration levels and their propagation during running.

## 3. Experimental Results

### 3.1. MTws Dynamic Calibration

**Figure 4:**
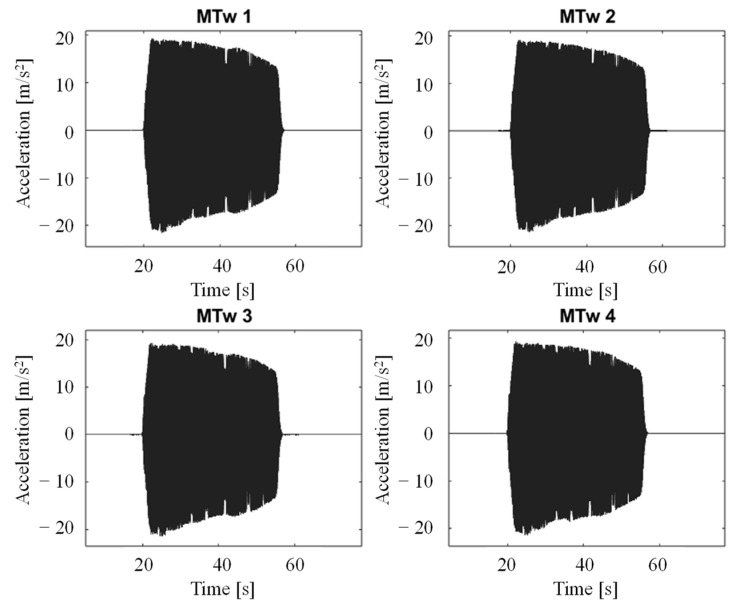
MTw acceleration time histories in the Z direction, 2 *g* sweep sine test in the dynamic calibration testing.

**Figure 5:**
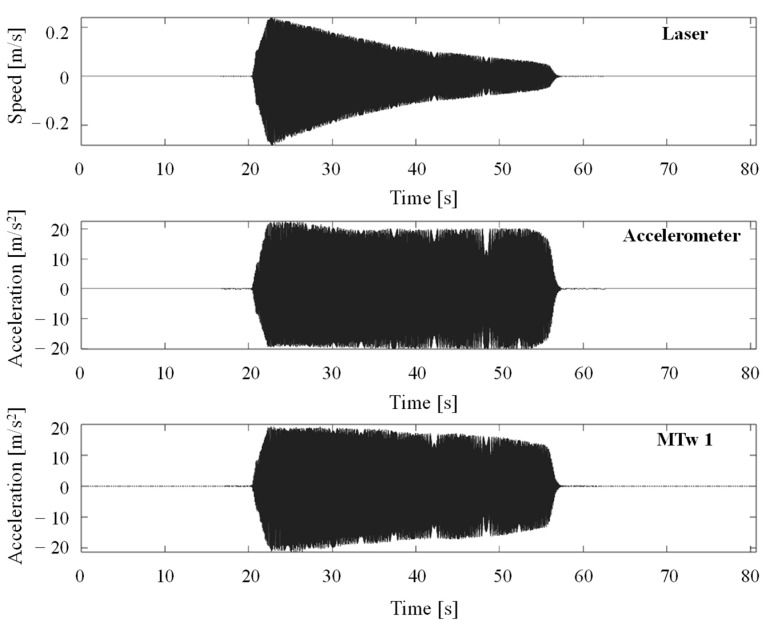
Synchronisation of acquired signals in the time domain for the 2 *g* sweep sine test. (Top) laser vibrometer, (middle) piezoelectric accelerometer, (bottom) MTW 1 acquired data.

Figure 4 and Figure 5 show acquired time histories by the MTws, the vibrometer and the piezoelectric accelerometer of the shaker head during the “2 *g*” sweep sine test.

Angle stability of the MTws was assessed by measuring the pitch, yaw and roll angles. Table 3 summarises the worst-case stability found during the MTw dynamic calibration.

**Figure 6:**
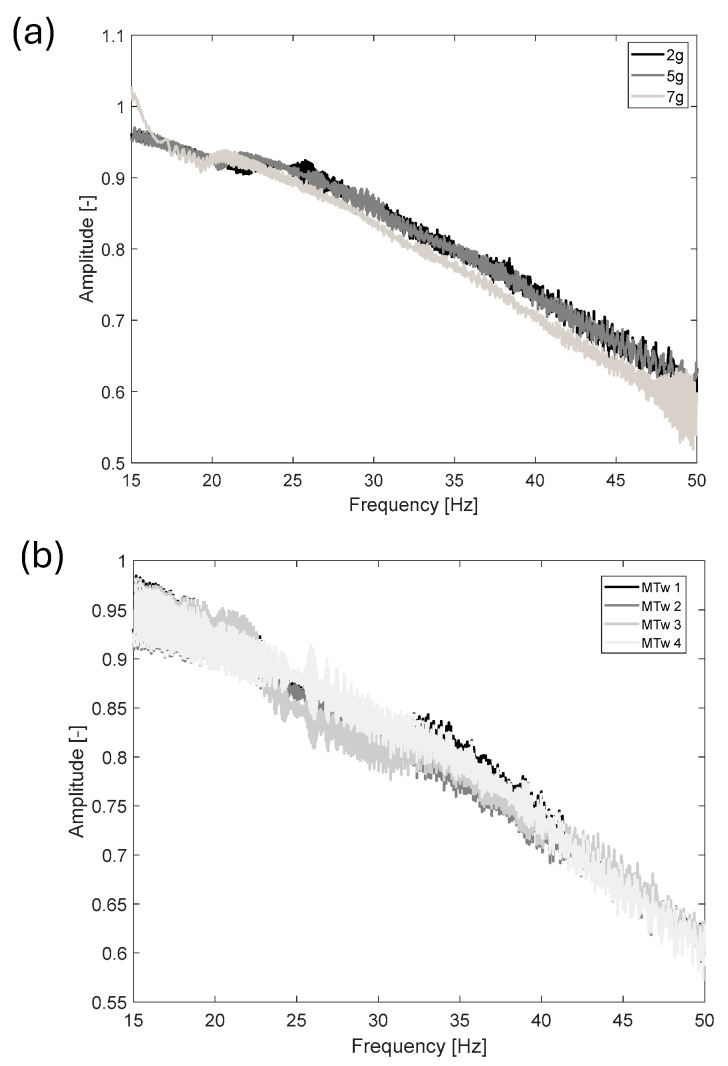
Measured FRF between the MTws and the laser vibrometer: (**a**) *H_1_* amplitude for MTw 3 for different acceleration levels (z-axis); (**b**) *H_1_* amplitude for all tested MTws at the 2 *g* excitation level.

In Figure 6a, measured FRF amplitudes for MTw 3 are shown by changing the sweep sine excitation level from 2 *g* to 7 *g*, while Figure 6b provides the measured FRFs for each tested MTw (z-axis) with 2 *g* sweep sine excitation. The curves show the sensor’s dynamic behaviour along the z-axis, one of the three local reference directions, which is consistent across all axes of the tested MTws.

The measured *H_1_* amplitudes were fitted by a polynomial function to allow for dynamic correction of the raw acquired acceleration in running tests. Measured data were fitted by a fourth-order function in the frequency range between 0 and 55 Hz. Table 4 summarises the results of the performed regression and the coefficient of the polynomial function and the related uncertainties; the latter values were computed with a 68% confidence bound.

### 3.2. Running Tests—Vibration Transmissibility and Exposure Assessment

**Figure 7:**
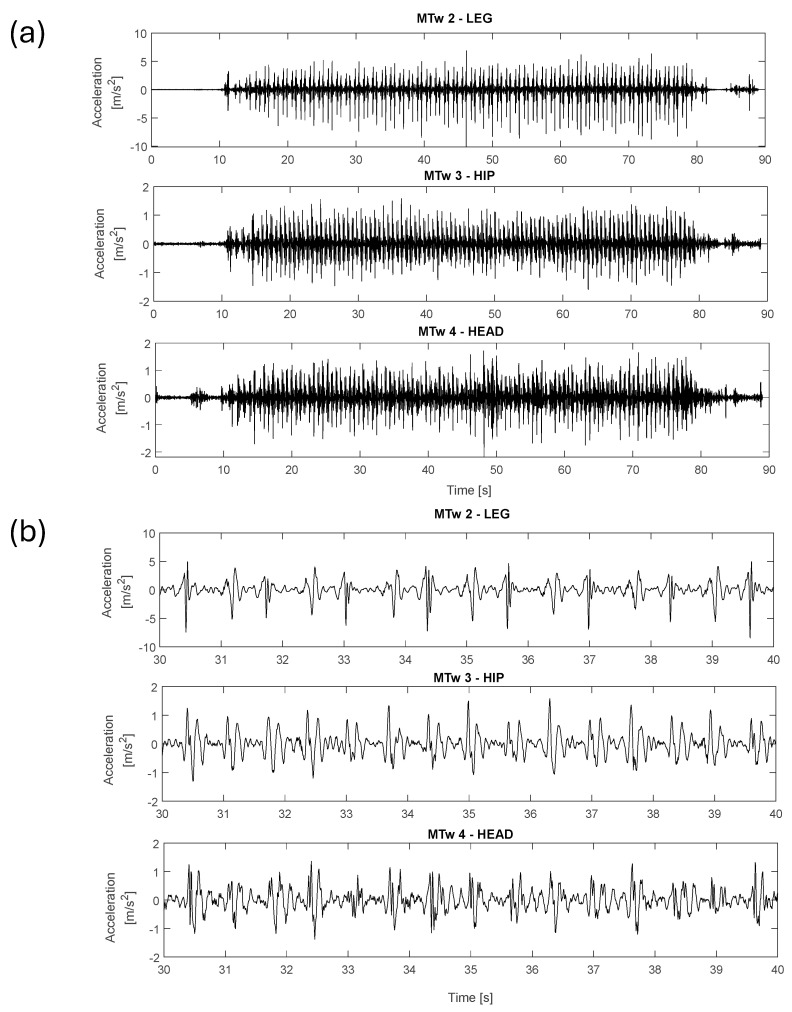
High-pass filtered acceleration signals along the Z-axis recorded during Test 0 (walking): (**a**) the entire time history; (**b**) a detailed view of 10 s timeframe.

**Figure 8:**
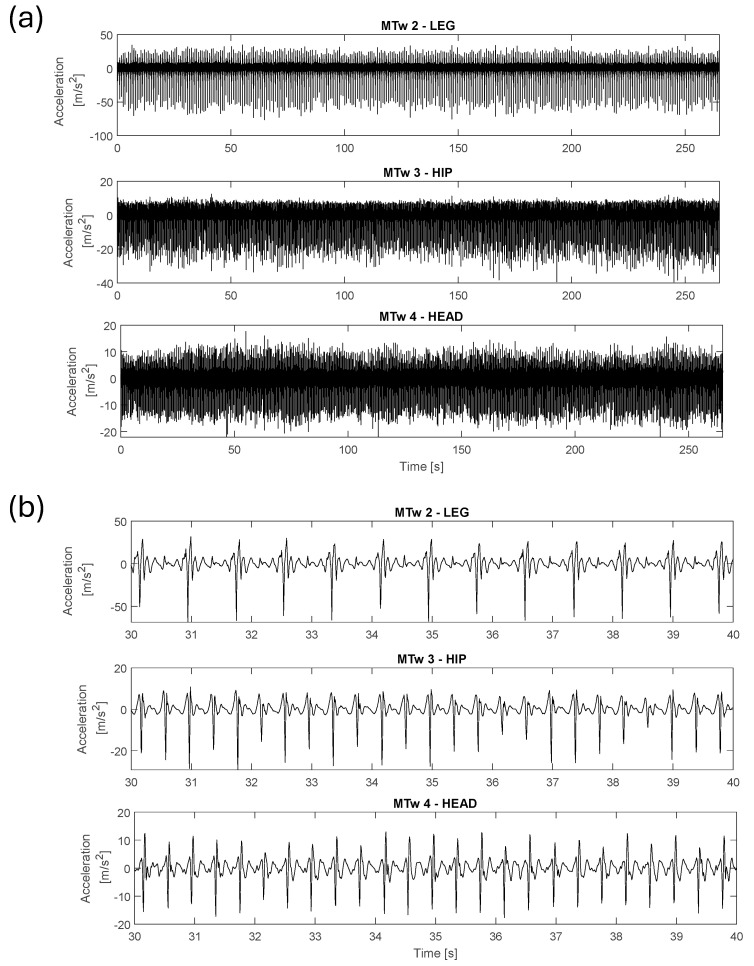
High-pass filtered acceleration signals along the Z-axis recorded during Test 1 (running at 8 km/h): (**a**) entire dataset; (**b**) detailed view for 10 s timeframe.

Figure 7 and Figure 8 show the acquired data along the vertical direction (ISO Z-axis) during the vibration level assessment testing, showing the time-domain signals recorded by the MTws. Two cases are presented: the first one describes Test 0 (walking), whereas the second one shows Test 1 (slow-speed running). Each figure includes the full dataset in panel (a) and a 10 s detailed view-in segment in panel (b).

**Figure 9:**
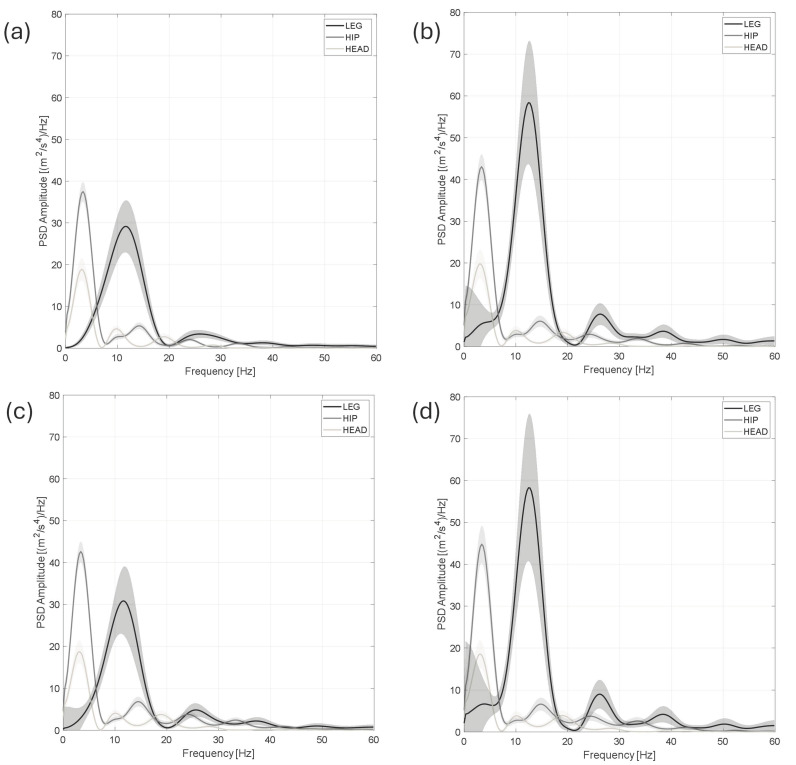
Comparison of the measured PSDs for the different tests: (**a**) Test 1, (**b**) Test 2, (**c**) Test 3 and (**d**) Test 4. Black, dark grey, and light grey show the average PSD amplitudes for the sensor mounted on the leg, hip and head, respectively; 1σ uncertainty bands are shown as filled trends in grey colour.

Furthermore, the PSDs computed in the running tests are shown in Figure 9 with related 1σ uncertainty bands.

**Figure 10:**
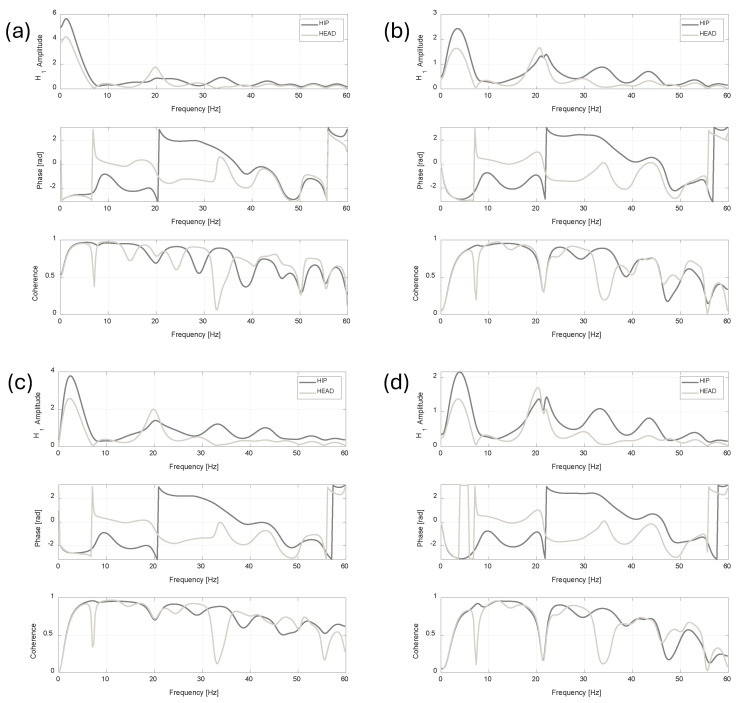
Vibration transmissibility assessment. The *H_1_* FRF estimator is computed considering MTw2 (LEG) as input and MTw3 (HIP) and MTw4 (HEAD) as outputs. *H_1_* estimators for Test 1, Test 2, Test 3 and Test 4 are shown in (**a**), (**b**), (**c**) and (**d**), respectively.

Figure 10 summarises computed *H_1_* estimators for each tested case in the vibration transmissibility analysis.

**Figure 11:**
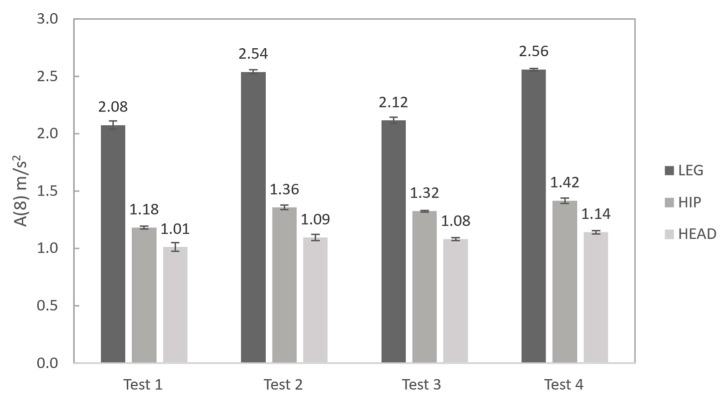
Comparison between the average *A*(8) measured values and related variability (1σ uncertainty bars) for the running tests.

The *a_w_* values were evaluated for each test, as shown in Table 5, Table 6, Table 7 and Table 8. Table 9 summarises the ratios of the weighted RMS along the X and Y directions over the Z direction to highlight their contributions to the overall measured *a_w_* for each tracked position. It has to be remembered that *a_w_* was computed considering four subsets, each lasting 30 s, for every tested condition. This allowed assessing repeatability during the testing of the computed metric. Moreover, a comparison between the averages of the measured *A*(8) values is given in Figure 11, considering 10 min exposure time over the reference time of 8 h.

## 4. Discussion

Dynamic calibration of the tested MTws showed that a correction is mandatory if acceleration outputs from the sensors have to be used for time domain and frequency domain analyses. In fact, as shown in Figure 4, the acceleration levels measured by the MTws showed a reduced amplitude in time, even when a constant acceleration amplitude was applied to the shaker head. Monitoring of the measured angle of the MTws showed, in the worst-case scenario (Table 3), negligible variations during dynamic calibration, validating the fixation method of the sensors on the shaker head and excluding any possible orientation effects on the measured FRF. By computing *H_1_* (FRF estimator) in all the tested cases after resampling and synchronisation (Figure 5), a general reduction of about 40% of the measured amplitude with respect to the input one was found. The maximum reduction was located at the extreme of the investigated frequency range, i.e., about 55 Hz, as shown by Figure 6a, where the comparison of the measured *H_1_* amplitudes at the different tested sweep sine amplitudes (from 2 *g* to 7 *g*) is given. It can be noticed that the measured attenuation is independent of the input vibration level, as shown by the overlapping of the FRF amplitude varying the input excitation. This highlights that the behaviour of the tested sensors is linear within the investigated sweep sine amplitude range. This result is of paramount importance because it stresses the need to implement a proper dynamic correction procedure to measure the right amplitude acceleration values in the entire bandwidth of the used sensors. In fact, if the correction procedure is not implemented, as was done for the present case study, an underestimation of the acceleration levels up to about 40% may occur, considering the frequency content near the bandwidth of the MTws. The cause of the reduction can be attributed either to a mechanical or electrical filtering of the MTws, which, being MEMS transducers, show this general behaviour in the frequency domain [39].

Given the obtained results, the definition of a single compensation curve is a feasible solution for the used sensors and provides a viable and simple approach for correcting the running tests, even for potential on-field applications. Eventually, the measured attenuation was found to be repeatable, considering that different MTws showed similar behaviour, as shown by the attenuation trends reported in Figure 6b. Table 4 summarises the coefficients of the derived 4th-order polynomial used for the dynamic correction. The measurement relative uncertainty related to the amplitude correction for the performed polynomial regression, derived from the sum of the squared residuals (SSRs), was found to be about 7.8%, a value considered accurate enough for the subsequent vibration transmissibility and exposure analyses.

Analysing running test results, it was found that the measured vibration is characterised by impulsive repetitive trends. As expected, vibration amplitudes increase during running, which can be seen by comparing the measured acceleration amplitudes along the vertical direction (Z axis) during walking (test 0) and running (test 1), as shown in Figure 7 and Figure 8. Focusing on the acceleration levels in Figure 8b, it can be highlighted that the amplitudes of the impulses reduce from the leg to the head by about a factor of 5. This vibration reduction was confirmed by the performed analyses in the frequency domain (PSD evaluation), provided in Figure 9 for each tested condition. In the tests with the faster pace (tests 2 and 4), the measured PSDs—averaged over about 160 zero-padded impulses for each test—showed the highest amplitudes compared to those measured at the hip or at the head, as shown in Figure 9b,d. This gap reduces for the slowest pace (i.e., in tests 1 and 3), as shown in Figure 9a,b, where the amplitudes of PSDs become compatible while those measured on the leg exhibit the largest frequency ranges in which the excitation of the impulse is present. The latter result justifies why the measured RMS at the leg is still the highest vibration contribution compared to the others, as found in the vibration exposure analyses.

Moreover, as shown by the measured PSD trend, the highest spectral amplitudes were found in the range between 5 and 20 Hz, in agreement with previous literature studies [20,40]; it is important to note that the PSD maxima are located at different frequencies, i.e., at about 5 Hz and 12 Hz, for the hip/head and leg, respectively. This was confirmed by the transmissibility analysis, based on *H_1_* estimator computation, shown in Figure 10. Given the different frequency distribution of the measured vibration on the hip and on the head, the measured FRFs show amplification at about 5 Hz compared to the leg, with a factor ranging from 2 to 5.5 for the hip and 1.5 to 4 for the head. In addition, the highest spectral amplitudes were found in the range between 5 and 20 Hz, in agreement with the literature [20,40].

Moreover, computed *H_1_* showed acceptable coherence, generally over 0.6 for the entire frequency range.

Despite transmissibility analysis, as previously mentioned, general vibration reduction was found from the leg to the hip and/or the head. This was confirmed by assessing weighted RMS acceleration levels, along X, Y and Z directions, at the measured positions. In fact, computed *a_w_* values from the MTw mounted on the leg (MTw2) showed the highest amplitudes in all the tested conditions, as highlighted in Table 5, Table 6, Table 7 and Table 8. The average *a_w_* values measured at the hip and at the head were lower than those at the leg by a factor ranging between 40% and 60%, depending on the analysed condition. Moreover, repeatability of the measured *a_w_* was good, with a worst-case relative uncertainty of about 3.7% of the average value (in particular for the MTw on the head, test 1, Table 5). Analysing the *a_w_* trend in time, i.e., comparing the measured values for the 2 min timeframe of the analysis, no significant variation was found, and therefore, no fatigue effect was highlighted within the duration of the test.

Detailing the contribution of each axis on the overall measured *a_w_*, as summarised in Table 9, it can be seen that the vertical direction is dominant, in particular for the hip location; at other positions, contributions from the other axes to the overall vibration also increase, reaching roughly equal weight in overall measured amplitude for the MTw2, positioned on the leg, in the fastest pace tests.

Assessment of the vibration exposure based on *A*(8) computation on 10 min exposure indicated that running speed had a significant influence on the measured values: MTw2 (on the leg) showed the highest values, providing *A*(8) amplitudes about 20% greater in the fast-paced running tests. A similar conclusion was found for the hip, but the amplitude increase, ranging between 5% and 15%, depended on the analysed condition. In the case of the head measurements, no significant variations were detected, providing relatively stable values within the tested conditions. Eventually, it can be seen that the comfort threshold for whole-body vibration exposure (set by the ISO standard at 2 m/s^2^) can be achieved for the tested subject with a time of about 10 min activity, as shown in Figure 11.

### Limitations and Future Developments

The findings of the research have important limitations that must be noted: (1) The induced vibration level assessment during running was preliminary, based on a single subject, so the computed metrics cannot be considered statistically robust; indeed, an extended campaign should be performed by increasing the number of involved subjects and testing cases. (2) The running tests did not adhere to a standardised protocol because no gold-standard reference system was available. Future studies should address this limitation by establishing repeatable and reproducible testing conditions across different athletes, in line with the procedure proposed in this work. (3) The applicability of the ISO 2631 approach to assess comfort and/or related RRIs is still debated in the scientific community, given the nature of the investigated physical phenomenon, characterised by subsequent impulses with the ground and not by a vibration transmitted by the ground to the human body. Nevertheless, no universally accepted standard exists for quantifying impulsive whole-body vibrations in running; therefore, the measured metric can be considered as a starting point for future research activities.

Despite these limitations, this research contributes preliminary insight into vibration exposure in running, providing a quantitative assessment of vibration transmissibility and levels. It shows that the tested sensors are suitable to accurately monitor induced vibrations during running, although prior dynamic correction for their behaviour in frequency is required.

Future developments of this study will focus on the symmetry between the right and left legs during the activity, comparing how the symmetry of the gesture is affected by fatigue and by changes in the running speed. Moreover, applicability to amateur runners will be investigated, thanks to the portability of the measurement system, in order to characterise the vibration environment in uncontrolled environments, to assess vibration dose levels, and to highlight possible risks of exposure in such conditions.

## 5. Conclusions

The assessment of using wearable devices, i.e., inertial measurement units (IMUs), for the investigation of vibration response and exposure during running was successfully carried out in this work.

The sensors’ bandwidth was characterised, highlighting the need for dynamic compensation to properly measure the acceleration levels in both the time and frequency domains. The calibration procedure involved applying different levels of vibration, each repeated twice, to four sensors tested under varying excitation levels. The results were repeatable and reproducible, with sensor performance decreasing by approximately 40% at the upper end of the acquisition bandwidth. Hence, this study highlights the need to implement a proper dynamic correction procedure to measure the correct acceleration amplitude values across the bandwidth of the sensors. A correction function was therefore derived, showing low relative measurement uncertainty, set to about 7.8%.

The research outcome highlights the need to implement a proper dynamic correction procedure whenever MTws are used to assess vibration dose exposure, e.g., in industrial scenarios or in sports activities involving whole-body vibration.

The proposed correction was applied to running tests performed in a controlled environment (treadmill) at different speeds. The obtained results are consistent with the literature, showing progressive attenuation of the vibration levels from the leg to the head and an increase in the vibration levels with increasing running speed.

The data processing involved several important analyses. The FFT was calculated for each subset, and in the frequency domain, dynamic correction and ISO 2631 weighting curves were applied for each vibration direction. From the weighted spectra obtained, RMS acceleration values were calculated. Moreover, assessment of the vibration dose using the *A*(8) metric (ISO-2631) showed that the threshold vibration dose for comfort awareness can be easily achieved within tens of minutes at the highest running speed (11 km/h). In particular, the preliminary assessment indicated that running speed has a significant influence on the measured values; the accelerations measured on the subject’s leg showed the highest values, providing *A*(8) amplitudes approximately 20% higher in fast-paced running tests. A comparable pattern was observed at the hip; however, the increase in amplitude—ranging from 5% to 15%—varied depending on the condition analysed. In contrast, measurements taken at the head showed no significant changes, indicating relatively stable values across the tested conditions.

Despite the limitations of the research, which need to be addressed by extending the experiment and demonstrating that the used metric can be correlated to RRIs, the main finding is that the investigated sensors are capable of measuring vibration levels during running, extending their applicability to indoor and outdoor scenarios, thanks to the portability and ease of implementation of the measurement system. In addition, the study paves the way for future research and applications, with the methodology applicable to a larger number of participants. It is also capable of measuring on-field vibration exposure during running for athletes with disabilities, thus enabling a comprehensive vibration assessment and robust statistical analysis during the intended activity.

## Figures and Tables

**Table 1:** Xsens Motion Trackers Awinda characteristics.

Specification	Performance
Full-Scale acceleration (FS)	±160 m⸱s^−2^
Non-linearity	0.5% of FS
Bias stability *	0.1 × 10^−3^ *g* **
Noise	200 µg/Hz^0.5^
Alignment error	0.1°
Bandwidth	180 Hz

* Allan variance diagram. ** The *g* is meant to be the Earth’s gravitational acceleration.

**Table 2:** MTw motion tracker technical specifications.

Specification	MTw Motion Tracker	Awinda Station
Communication interface	Wireless 2.4 GHz/USB	Wireless 2.4 GHz/USB
Wireless transmit range indoor/outdoor	~20 m/70 m	~50 m/20 m

**Table 3:** Measured angles by MTws—worst-case scenario during dynamic calibration.

Angles ***	Mean Value [°]	Standard Deviation [°]
Roll ϕ (x)	−179.69	0.05
Pitch θ (y)	0.20	0.05
Yaw ψ (z)	−98.49	0.11

*** Local Reference System.

**Table 4:** Polynomial correction curve coefficients, standard deviation at 68% confidence bound, degrees of freedom (DFs), and sum of the squared residuals (SSRs).

Parameter	Units	Value	1σ Uncertainty
*a*_4_	Hz^−4^	3.78 × 10^−7^	±2.9 × 10^−8^
*a*_3_	Hz^−3^	3.79 × 10^−5^	±3.3 × 10^−6^
*a*_2_	Hz^−2^	1.04 × 10^−3^	±1.2 × 10^−4^
*a*_1_	Hz^−1^	−1.18 × 10^−2^	±1.3 × 10^−3^
*a*_0_	-	1	n.d.
*DF*	-	4796	
*SSR*	-	29.3	n.d.

**Table 5:** *a_w_* values (m/s^2^ units) for Test 1.

	Test 1 _1	Test 1 _2	Test 1 _3	Test 1 _4	Average	1σ
MTw2—Leg	14.24	14.69	14.44	14.14	14.38	0.24
MTw3—Hip	8.15	8.31	8.20	8.10	8.19	0.09
MTw4—Head	6.73	7.27	7.18	6.83	7.00	0.26

**Table 6:** *a_w_* values (m/s^2^ units) for Test 2.

	Test 1 _1	Test 1 _2	Test 1 _3	Test 1 _4	Average	1σ
MTw2—Leg	17.55	17.57	17.47	17.77	17.59	0.13
MTw3—Hip	9.26	9.30	9.45	9.56	9.39	0.14
MTw4—Head	7.43	7.45	7.63	7.83	7.58	0.19

**Table 7:** *a_w_* values (m/s^2^ units) for Test 3.

	Test 1 _1	Test 1 _2	Test 1 _3	Test 1 _4	Average	1σ
MTw2—Leg	14.48	14.54	14.79	14.87	14.67	0.19
MTw3—Hip	9.20	9.12	9.14	9.23	9.17	0.05
MTw4—Head	7.37	7.48	7.51	7.58	7.48	0.09

**Table 8:** *a_w_* values (m/s^2^ units) for Test 4.

	Test 1 _1	Test 1 _2	Test 1 _3	Test 1 _4	Average	1σ
MTw2—Leg	17.64	17.77	17.68	17.82	17.73	0.08
MTw3—Hip	9.60	9.75	9.92	9.95	9.81	0.16
MTw4—Head	7.78	7.91	8.04	7.85	7.90	0.11

**Table 9:** Ratio of the weighted RMS along X and Y directions over Z direction, for each sensor and in each running test.

	MTw2 (LEG)		MTw3 (HIP)		MTw4 (HEAD)	
	RMS_X_/RMS_Z_	RMS_Y_/RMS_Z_	RMS_X_/RMS_Z_	RMS_Y_/RMS_Z_	RMS_X_/RMS_Z_	RMS_Y_/RMS_Z_
Test 1	0.73	0.67	0.18	0.20	0.28	0.55
Test 2	0.77	0.90	0.27	0.18	0.29	0.60
Test 3	0.73	0.68	0.17	0.21	0.31	0.60
Test 4	0.78	0.90	0.17	0.27	0.34	0.66

## Data Availability

The data presented in this study are available upon request from the corresponding author.

## Abbreviations

The following abbreviations are used in this manuscript:

RRIs	Running-Related Injuries
IMU	Inertial Measurement Unit
MTw	Xsens Motion Trackers Awinda
MEMS	Micro-ElectroMechanical Systems
FFT	Fast Fourier Transform
FRF	Frequency Response Function
PSD	Power Spectral Density
